# To Clot or Not to Clot: Is That the Question?

**DOI:** 10.3390/jcm12062381

**Published:** 2023-03-19

**Authors:** Emmanuel J. Favaloro

**Affiliations:** 1 Haematology, Institute of Clinical Pathology and Medical Research (ICPMR), Sydney Centres for Thrombosis and Haemostasis, NSW Health Pathology, Westmead Hospital, Westmead, NSW 2145, Australia; emmanuel.favaloro@health.nsw.gov.au; 2 School of Dentistry and Medical Sciences, Faculty of Science and Health, Charles Sturt University, Wagga Wagga, NSW 2650, Australia; 3 School of Medical Sciences, Faculty of Medicine and Health, University of Sydney, Westmead Hospital, Westmead, NSW 2145, Australia

Hemostasis can be defined as a homeostatic process in which the body attempts to minimize loss of blood by balancing out pro- and anti-procoagulant forces. Getting the balance right is easier in health than it is when the body is subjected to a disease process. When anti-coagulant forces dominate, bleeding can ensue, and when pro-coagulant forces dominate, thrombosis may occur and ultimately block blood flow through a particular blood vessel, which can then lead to tissue death or organ failure.

There are several examples of diseases that lead to thrombosis. One of the most contemporary examples is the Coronavirus disease 2019 (COVID-19), which can arise after infection with the severe acute respiratory syndrome Coronavirus 2 (SARS-CoV-2). Although primarily a lung infection, severe disease can lead to a condition called COVID-19-associated coagulopathy (CAC), which essentially reflects a pro-thrombotic syndrome [1]. This largely inflammation-driven condition leads to a state of continued coagulation activation, including the release of pro-hemostatic proteins, such as von Willebrand factor (VWF), which can overcome the ability of the body’s normal regulatory system, including the VWF cleaving protease ADAMTS13 (a disintegrin and metalloproteinase with thrombospondin type 1 motif, 13). This leads to an imbalance of the VWF/ADAMTS13 axis, and potentially to (micro-)thrombosis and end organ damage/failure [2]. To help prevent thrombosis in moderate-to-severe COVID-19, it is recommended that patients be given anticoagulant therapy, and potentially antiplatelet agents. The most common anticoagulant given is heparin [3], and the most common antiplatelet agent potentially applied is aspirin [4]. Heparin is a common anticoagulant given to treat or prevent venous thrombosis, and anti-platelet agents such as aspirin are commonly administered to help prevent arterial thrombosis. Interestingly, the benefits of heparin anticoagulant in the prevention of thrombosis in COVID-19 appear more conclusive than the benefits of anti-platelet agents such as aspirin [4]. Similar controversial results are also evident with other antiplatelet agents such as the P2Y12 inhibitors, including clopidogrel, or with the use of dual antiplatelet therapy (e.g., aspirin plus clopidogrel). Indeed, as with all situations in which such compounds are used, there is a need to balance the risk of thrombosis (without the compound(s)) versus the risk of bleeding (with the compound(s)). Another interesting perspective here relates to how particular agents are used. For heparin, there are a variety of formulations, including both unfractionated and low molecular weight varieties. Heparin is usually given parenterally (i.e., injected subcutaneously or intravenously). However, there is increasing evidence that heparin has additional benefits beyond that of anticoagulation, and for example, a local anti-inflammatory effect and potential to reduce SARS-CoV-2 binding (and thus subsequent infection) is evident when heparin is applied intra-nasally [3].

Another example of a pro-coagulant disease is antiphospholipid antibody syndrome (APS), which arises due to the formation of antiphospholipid antibodies (aPL). These antibodies can arise against a variety of phospholipid moieties [5]. Perhaps unsurprisingly, given the above context, patients with APS, reflecting an autoimmune disorder, are also given anticoagulant therapy to help treat and prevent thrombosis. Again, the use of both aspirin and heparin may play a role in these patients [5,6], but also recognizing that prevention of thrombosis may come with the risk of bleeding. For the long-term prevention of venous thrombosis, APS patients are often placed on vitamin K antagonist (VKA) therapy, such as warfarin. In such long-term management, the risk of bleeding increases, and patients are maintained within narrow therapeutic ranges using a common laboratory test called the INR (international normalized ratio). Of course, APS is just one of a plethora of auto-immune conditions associated with hemostatic imbalance, primarily manifesting as thrombosis [7]. Additional examples of auto-immune conditions leading to thrombosis are heparin-induced thrombocytopenia with thrombosis (HITT) and vaccine-induced thrombocytopenia with thrombosis (VITT) [7,8]. This leads me to the concept of the ‘inter-connectedness of all things’, given that an agent normally given to prevent thrombosis (heparin) can in a small number of susceptible individuals lead instead to thrombosis, or that vaccination against COVID-19 to help prevent the adverse events of serious SARS-CoV-2 infection, including thrombosis, can itself instead lead to thrombosis in an even smaller number of susceptible individuals. To provide context here, HITT will occur in less than 5% of patients administered heparin, and VITT will only occur in some 1 in 100,000 immunised individuals [8]. In contrast, heparin will prevent thrombosis, and immunization will prevent COVID-19 in the vast majority of individuals given these therapies. Nevertheless, such adverse possibilities reflect the balance that hemostasis tries to deliver when faced with a disease process. Yet other interesting interconnections are represented by the fact that both aPL and HITT-like antibodies have been described as a consequence of COVID-19 [8,9].

Another final example of increased thrombosis risk associated with the disease that I will provide is the state of cancer [10,11]. For example, in cancer patients, pulmonary embolism (PE) is the second leading cause of death after cancer itself, possibly because of difficulties in diagnosing the disease due to its nonclassical presentation [10]. Like the other conditions associated with an increase in thrombosis, anticoagulation plays a central role in the management of patients with cancer and PE, as well as helping to prevent further thrombosis. However, managing venous thromboembolism (VTE) in cancer patients is more challenging than that of noncancer patients, because of the frail balance between the increased risk of recurrent VTE and the increased risk of major bleeding due to the anticoagulant treatment [10].

The other side of the hemostasis coin, when things do not go according to plan, is bleeding. Bleeding can arise in a large variety of associated conditions. There are a large number of congenital and acquired bleeding disorders. I previously mentioned that the risk of thrombosis when the VWF/ADAMTS13 axis is driven towards an excess of VWF, may, for example, occur in severe COVID-19 [2]. Unfortunately, too little plasma VWF can instead lead to bleeding, with this condition arising in the common congenital bleeding disorder called von Willebrand disease (VWD) or in acquired von Willebrand syndrome (AVWS) [12]. Regarding the interconnectedness of all things, VWF and ADAMTS13 have intriguing connections with both cancer and cardiac disease that go beyond those of bleeding or thrombosis risk [11]. For example, VWF may assist certain cancers to spread or metastasize [11]. In terms of cardiac disease, VWF may be implicated in both increased bleeding risk and increased thrombotic risk [13], depending on the type of cardiac disease and the plasma level and activity of VWF. If there is a risk of thrombosis with particular cardiac disease processes, then anticoagulant or other anti-thrombotic therapy may be applied [14,15,16].

Another common bleeding disorder is hemophilia A, representing a loss of coagulation factor VIII (FVIII) activity. There are a variety of potential therapies applied to help prevent bleeding in these patients, including FVIII replacement therapy [17]. Because of the high risk of development of antibodies against the infused replacement FVIII, rendering such therapy ineffective, alternate ‘bypass’ therapies may also or alternatively be applied. One contemporary novel treatment is a compound called Emicizumab [18]. This compound is a humanized IgG4 bi-specific antibody with affinity to factor IX/FIXa and factor X, and thus mimics the co-factor activity of FVIII by bridging the two factors and promoting coagulation independent of FVIII.

The final example of inter-connectiveness that I will give in this editorial is that of laboratory testing. The modern pathology laboratory can perform a large variety of tests, both related to hemostasis and otherwise. The laboratory can test for most of the components mentioned above. This includes heparin, VWF, ADAMTS13, FVIII, Emicizumab, SARS-CoV-2 infection, HITT, and VITT. This also includes the use of potential test panels for certain disease states, such as COVID-19 [19,20].

In conclusion, the process of hemostasis attempts to minimize the loss of blood by balancing out pro- and anti-coagulant forces. In certain disease states, the process can go awry, and then lead to either excessive bleeding or thrombosis. Clinicians can treat these disorders and attempt to rebalance hemostasis. In bleeding disorders such as VWD, AVWS and hemophilia A, the main therapies are factor replacement or bypass therapies. Here, there is a risk of giving ‘too much’ procoagulant material, and thus a potential for thrombosis, but this risk can be mitigated by laboratory testing of the replacement material or its effects on hemostasis to ensure maintenance of physiological levels, rather than given rise to pathological levels. In the case of pro-thrombotic disorders, such as COVID-19, APS, or atrial fibrillation, then clinicians will largely apply anticoagulant or other anti-thrombotic therapy (e.g., anti-platelet agents). Here, there is a risk of giving ‘too much’ anticoagulant material, and thus potential bleeding risk. Again, the pathology laboratory may be able to mitigate the bleeding risk by laboratory testing of assessment of anticoagulant therapy effects, although except for VKA therapy monitoring, this would be on an exception basis rather than the norm. Irrespective, when hemostasis goes awry in certain disease states, it can be rebalanced, but this does come at a risk, should the rebalance tip the balance too far towards the alternate direction. Such is the rich tapestry of hemostasis. To clot or not to clot—that may indeed be the question.
